# Exercise volume and aerobic fitness in young adults: the Midwest Exercise Trial-2

**DOI:** 10.1186/s40064-016-1850-0

**Published:** 2016-02-25

**Authors:** Matthew M. Schubert, Richard A. Washburn, Jeffery J. Honas, Jaehoon Lee, Joseph E. Donnelly

**Affiliations:** Division of Internal Medicine, Cardiovascular Research Institute, The University of Kansas Medical Center, Kansas City, KS USA; Department of Kinesiology, Auburn University at Montgomery, Montgomery, AL USA; University of Kansas, Robinson Center Rm. 100, 1301 Sunnyside Avenue, Lawrence, KS 66046 USA; Institute for Measurement, Methodology, Analysis, and Policy, Texas Tech University, Lubbock, TX USA

**Keywords:** Cardiovascular fitness, Intensity, Supervised exercise, Overweight

## Abstract

To examine the effect of exercise volume at a fixed intensity on changes in aerobic fitness. Ninety-two overweight/obese individuals (BMI 25–40 kg m^2^), age 18–30 years, 50 % women, completed a 10 mo, 5 d wk^−1^ supervised exercise intervention at 2 levels of exercise energy expenditure (400 or 600 kcal session^−1^) at 70–80 % heart rate (HR) max. Exercise consisted primarily of walking/jogging on motor-driven treadmills. The duration and intensity of all exercise sessions were verified by a downloadable HR monitor set to collect HR in 1-min epochs. All participants were instructed to continue their typical patterns of non-exercise physical activity and dietary intake over the duration of the 10 mo intervention. Maximal aerobic capacity (indirect calorimetry) was assessed on a motor-driven treadmill using a modified Balke protocol at baseline, mid-point (5 mo), and following completion of the 10 mo intervention. VO_2_ max (L min^−1^) increased significantly in both the 400 (11.3 %) and 600 kcal session^−1^ groups (14 %) compared to control (−2.0 %; *p* < 0.001); however, the differences between exercise groups were not significant. Similar results were noted for change in relative VO_2_ max (mL kg^−1^ min^−1^); however, the magnitude of change was greater than for absolute VO_2_ max (L min^−1^) (400 group = 18.3 %; 600 group = 20.2 %) due to loss of body weight over the 10-mo intervention in both exercise groups. Our results indicate that exercise volume was not associated with change in aerobic fitness in a sample of previously sedentary, overweight and obese young adults.

## Background

Low aerobic fitness is associated with increased risk for all-cause and cardiovascular disease mortality (Berry et al. 2011; Gander et al. 2011; Shuval et al. 2012) as well as the risk for several chronic conditions including diabetes (Wei et al. 1999; Goodrich et al. 2012; Farrell et al. 2010), hypertension (Lee et al. 2012; Fogelhom 2010), cancer (Byun et al. 2011; Peel et al. 2009; Farrell et al. 2010), and dementia (Liu et al. 2012; Ahlskog et al. 2011). Higher levels of aerobic fitness confer health benefits irrespective of body weight, which may be potentially important since 69 % of the US population is currently overweight or obese (Ogden et al. 2014). For example, aerobic fitness has been associated with reduced mortality risk (Sui et al. 2007; Faselis et al. 2012; McAuley et al. 2010, 2012) and reduced risk for development of hypertension, metabolic syndrome and hypercholesterolemia (Lee et al. 2012) independent of obesity. Therefore, the effect of exercise prescription on changes in aerobic fitness in overweight and obese individuals represents an important clinical research question.

It is well established that when exercise volume is controlled, higher/vigorous intensity exercise [64–90 % VO_2_ max; 77–95 % heart rate (HR) max] (Garber et al. 2011) is superior to lower/moderate intensity exercise (46–63 % VO_2_ max; 64–76 % HR max) (Garber et al. 2011) for improving aerobic fitness (Swain 2005; Gormley et al. 2008; Helgerud et al. 2007; O’Donovan et al. 2005). However, when exercise intensity is controlled, the effect of exercise volume [prescribed as frequency and exercise energy expenditure (EEEx) or exercise time] on aerobic fitness is unclear. In a 2001 review on the total volume of physical activity and health and fitness Oja et al. (2001) concluded: “fairly strong evidence indicates a crude dose response between the total volume of weekly physical activity and cardiorespiratory fitness …”; however, it was noted that “None of the reviewed nonrandomized and randomized exercise trials have been designed to analyze specifically the dose response of the total exercise volume and health and fitness outcomes”. The results from the limited number of more recent randomized trials are mixed, with reports supporting (Church et al. 2007; Duscha et al. 2005; Dalleck et al. 2009) or failing to support (Duncan et al. 2005; Asikainen et al. 2002; Hautala et al. 2003; Rosenkilde et al. 2012) an association between exercise volume and change in aerobic fitness. Differences between study design, such as intervention length, method of prescribing exercise volume, level of exercise supervision, and age, gender, and baseline aerobic fitness of participants offer potential explanation for the discrepant results.

Data from the Midwest Exercise Trial-2 (MET-2) afforded an opportunity to examine the effect of exercise volume at a fixed intensity on changes in aerobic fitness in a sample of previously sedentary, overweight/obese young adults. The fact that MET-2 was a tightly controlled efficacy trial permitted us to examine a dose–response of exercise volume on aerobic fitness independent of intensity and frequency; thus providing some clarity on the discrepancies that exist in the literature regarding the associations between exercise volume and aerobic fitness. These results have potential public health policy utility as they will help identify the most appropriate exercise volume to achieve the greatest fitness gains, which is important as cardiovascular fitness is significantly associated with mortality risk (McAuley et al. 2010, 2012). The primary aims of MET-2 were to evaluate the role of aerobic exercise without energy restriction on weight and body composition; however, several secondary outcomes, including changes in aerobic fitness, were included a priori in the original study design.

## Methods

A detailed description of the design and methods for MET-2 (Donnelly et al. 2012), results for the primary aim (Donnelly et al. 2013), and additional secondary aims have been published previously (Willis et al. 2014; Washburn et al. 2015). Briefly, MET-2 randomized overweight or obese individuals (BMI 25–40 kg m^2^) aged 18–30 years to a 10 mo, 5 d wk^−1^ supervised exercise intervention at 2 levels of EEEx (400 or 600 kcal session^−1^) or non-exercise control. This trial was registered at ClinicalTrials.gov (NCT01186523) with primary outcome data collection occurring from July 2005 through July 2011.

### Participants

A total of 2338 individuals completed the on-line initial eligibility questionnaire from which 141 participants were randomized to one of the 3 study groups. Potential participants were excluded for the following reasons: A history of chronic disease (i.e. diabetes, heart disease, etc.), elevated blood pressure (>140/90 mmHg), lipids (cholesterol > 6.72 mmol L^−1^; triglycerides > 5.65 mmol L^−1^), or fasting glucose (>7.8 mmol L^−1^), use of tobacco products, taking medications that would affect physical performance (i.e., beta blockers, or metabolism (i.e. thyroid, steroids), inability to perform laboratory tests or participate in moderate-to-vigorous intensity exercise, and planned physical activity >500 kcal wk^−1^ as assessed by recall (Taylor et al. 1978). Participants provided written informed consent prior to engaging in any aspect of the trial and were compensated for participation. Approval for this study was obtained from the Human Subjects Committee at the University of Kansas-Lawrence and all research conformed to guidelines laid down in the Declaration of Helsinki.

### Randomization and blinding

Participants were stratified by gender and randomized by the study statistician (~80 % exercise; ~20 % control). All participants were instructed to continue their typical patterns of non-exercise physical activity and dietary intake over the duration of the 10 mo intervention. The blinding of participants to group assignment was not possible due to the nature of the intervention. However, both investigators and research staff were blinded at the level of outcome assessments, data entry and data analysis.

### Exercise training

Exercise, consisting primarily of walking/jogging on motor-driven treadmills, was supervised by trained research staff and conducted in a dedicated exercise facility in the Energy Balance Laboratory at the University of Kansas-Lawrence. To provide variety and decrease the potential for overuse injuries, alternate activities including stationary biking, walking/jogging outside, and the use of elliptical trainers was permitted for 20 % of the total exercise sessions (i.e., 1 session wk^−1^). The exercise protocol was designed to progress in intensity and amount from baseline to the end of month 4, both to provide time to adapt to exercise and prevent injuries.

The duration of exercise required to elicit either 400 or 600 kcal session^−1^ for each participant was determined as follows: At the baseline assessment, treadmill speed/grade was set at 3 mph/0 % grade and was adjusted by increments of 0.5 mph/1 % grade until the participant reached 70 % HR max (±4 beats min^−1^). Maximal HR was the highest HR rate achieved during the assessment of maximal aerobic capacity (described below). EEEx was then assessed over a 15 min interval (1-min epochs) using a ParvoMedics TrueOne2400 indirect calorimetry system (ParvoMedics Inc., Sandy, UT). The average EEEx (kcal min^−1^) over the 15 min interval was calculated from measured oxygen consumption and carbon dioxide production using the Weir equation (1949). This value was used to provide the goal for the duration of exercise sessions for the first month of the intervention. For example: prescribed EEEx during month 1 = 150 kcal session^−1^, EEEx = 9.2 kcal min^−1^, exercise duration = 150 kcal session^−1^ divided by 9.2 kcal min^−1^ = 16 min session^−1^. Similar procedures to determine exercise duration were conducted at the end of each month over the course of the 10 mo intervention to adjust for potential effects of changes in both body weight and aerobic fitness on EEEx. The duration and intensity of all exercise sessions were verified by a downloadable HR monitor (RS 400; Polar Electro Inc., Woodbury, NY) set to collect HR in 1-min epochs. All exercise sessions and assessments of EEEx were preceded by a brief warm up on the treadmill (~2 min, 3–4 mph, 0 % grade). Treadmill speed and grade were subsequently increased to achieve the prescribed target HR. Additionally, the level of perceived exertion (Borg 1982), treadmill speed and grade, and HR were recorded by the research assistant at 10 min intervals during each exercise session. This procedure provided interaction between study staff and participants and helped to maintain compliance, as well as providing a detailed description of each exercise session. Compliance to the exercise protocol, an essential element of an efficacy study, was defined as successfully completing >90 % of scheduled exercise sessions defined as maintaining the target exercise HR ± 4 beats min^−1^ for the prescribed duration of the exercise session. Participants who were non-compliant during any 3 mo interval (months 0–3, 3–6, 6–9) or during the final month (month 10) were dismissed from the study.

#### Control group

Participants assigned to the non-exercise control group were instructed to continue their typical patterns for physical activity and dietary intake over the duration of the 10 mo study. With the exception of assessment of EEEx, the same outcome assessments were completed with both the exercise and control groups.

### Outcomes

#### Aerobic fitness

Maximal aerobic capacity was assessed on a motor-driven treadmill using a modified Balke protocol (American College of Sports Medicine. ACSM’s guidelines for exercise testing and prescription 2009) at baseline, mid-point (5 mo), and following completion of the 10 mo intervention. Expired gases were collected in 20-s epochs (Parvo Medics n2400) and HR was monitored continually using a three lead electrocardiogram (Marquette Electronics, Milwaukee, WI, USA). Tests were considered valid if participants met three of the following four criteria: (1) HR ± 10 beats min^−1^ of the age-predicted maximal HR (220—age), (2) rating of perceived exertion (RPE) >17 on the 20-point Borg scale (1982), (3) respiratory exchange ratio (RER) >1.10, and (4) oxygen consumption plateau (i.e., no increase in oxygen consumption with increased workload) (Robergs et al. 2010). The maximal HR obtained was used for the determination of HR associated with the prescribed levels of EEEx as previously described.

### Other measures

Body weight was measured monthly using a digital scale accurate to ±0.1 kg (PS6600, Befour Inc., Saukville, WI). Energy intake was assessed at baseline, 3, 6, and 10 mo over a 7-d period of ad libitum eating in a University of Kansas cafeteria from digital photographs obtained before and after consumption (Hise et al. 2002; Grunwald et al. 2003). Daily physical activity was assessed by portable accelerometer (Actigraph Model GT1 M Actigraph, LLC, Pensacola, FL) over 7 consecutive days at baseline, 3, 6, and 10 mo.

### Statistical analysis

Sample size was determined to provide sufficient power for evaluating aerobic fitness in a design with one between-subjects factor (group: 400, 600 kcal, control) and one within-subjects factor (time: 3 levels, baseline, 5 and 10 mo). Our achieved sample size provided 85 % power to detect a medium difference (Cohen’s *f* = 0.25) in VO_2_ max (L min^−1^) among groups, with an alpha level of 0.05 and an assumed correlation among repeated measures as high as 0.60.

Sample demographics and all outcome measures were summarized by descriptive statistics; means and standard deviations for continuous variables and frequencies and percentages for categorical variables. Mixed modeling for repeated measures was used to estimate group differences in percent change in VO_2_ max, along with a proper error covariance structure that provided better model fit than other error covariance structures according to Akaike Information Criterion and Bayesian Information Criterion. When group effect or group x time interaction was significant at 0.05 alpha level, pairwise group means were compared using Bonferroni adjustment for inflated Type I error. In secondary mixed modeling analysis, the impacts of age, gender, baseline BMI and VO_2_ max, and study group and average exercise min session^−1^ were examined to identify the factors that may contribute to change in aerobic fitness. All analyses were conducted using SAS 9.3 (SAS Institute, Cary, NC).

## Results

### Participants

Ninety-two of the 141 participants randomized at baseline (65.2 %) complied with the study protocol and completed all outcome assessments. The completion rate was 75, 70 and 60 % for the control, 400 and 600 kcal session^−1^ groups, respectively. Approximately 44 % of those who did not complete the study were dismissed by the investigators for failure to comply with the study protocol. Additional reasons for drop out included lack of interest/time, schedule conflicts, and unwillingness to comply with the dietary assessment protocol. The baseline characteristics of the 92 participants who completed the study are presented in Table 1. The sample had a mean age of ~23 years, BMI ~ 31 kg m^2^, and was comprised of 50 % women. There were no differences in baseline characteristics between the 3 study groups or between participants who were initially randomized (n = 141) and those who completed the study protocol (n = 92) with the exception of a small but significantly higher level of aerobic fitness (*p* < 0.04) in participants who completed (33.4 ± 5.9 mL kg^−1^ min^−1^) versus those who did not complete the study protocol (31.4 ± 5.5 mL kg^−1^ min^−1^). No major adverse events were reported among participants in either the exercise or control groups.

**Table 1:** Baseline participant characteristics

	400 kcal session^−1^ *n* = 18 men, 19 women	600 kcal session^−1^ *n* = 19 men, 18 women	Control *n* = 9 men, 9 women
Age (years)	23.1 ± 3.0	23.0 ± 3.5	22.6 ± 3.0
Anthropometrics			
Weight (kg)	91.4 ± 20.7	92.0 ± 16.1	87.4 ± 14.6
BMI (kg m^2^)	31.2 ± 5.6	30.6 ± 3.9	29.7 ± 3.8
Race, No. (%)			
White	30 (83.3 %)	36 (97.3 %)	14 (77.8 %)
Other	6 (16.7 %)	1 (2.7 %)	1 (5.6 %)
Ethnicity No. (%)			
Not Hispanic or Latino	34 (94.4 %)	37 (100.0 %)	15 (83.3 %)
Exercise test variables			
Maximal HR (beats min^−1^)	196.8 ± 9.0	197.1 ± 8.7	195.8 ± 6.2
Respiratory exchange ratio	1.2 ± 0.0	1.2 ± 0.1	1.2 ± 0.1
Maximal VO_2_ (L min^−1^)	3.0 ± 0.7	3.1 ± 0.8	2.8 ± 0.6
Maximal VO_2_ (mL kg^−1^ min^−1^)	33.4 ± 6.5	34.1 ± 5.7	32.3 ± 5.0
Diet			
Energy intake (kcal d^−1^)	2887 ± 670	2948 ± 687	2836 ± 642

Unless otherwise stated values are mean ± standard deviation

### Exercise compliance

Data describing the exercise training intervention, excluding the initial 4 mo ramp-up period, are presented in Table 2. Attendance at exercise sessions was high (≥91 %) and did not differ by exercise group or gender. The average EEEx from month 4–10 for the 400 and 600 kcal session^−1^ groups was 402 ± 6 and 604 ± 7 kcal session^−1^, respectively. There were no differences in exercise intensity between exercise groups or between men and women assigned to exercise at 400 or 600 kcal session^−1^.

**Table 2:** Exercise intervention: descriptive information

	400 kcal session^−1^		600 kcal session^−1^	
	Men (n = 18)	Women (n = 19)	Men (n = 19)	Women (n = 18)
EEEx (kcal session^−1^)	401.1 ± 6.4	402.4 ± 7.5	605.2 ± 8.9	602.4 ± 11.7
Exercise time (min session^−1^)	31 ± 6	48 ± 7	42 ± 8	63 ± 9
Exercise intensity (% HR max)	78 ± 4	80 ± 2	80 ± 1	79 ± 3
Sessions attended	92 %	92 %	91 %	92 %

Values are mean ± standard deviation for months 4–10 of exercise training which excludes the 4 mo ramp-up period

*EEEx* exercise energy expenditure, *HR* heart rate

### Aerobic fitness

**Fig. 1:**
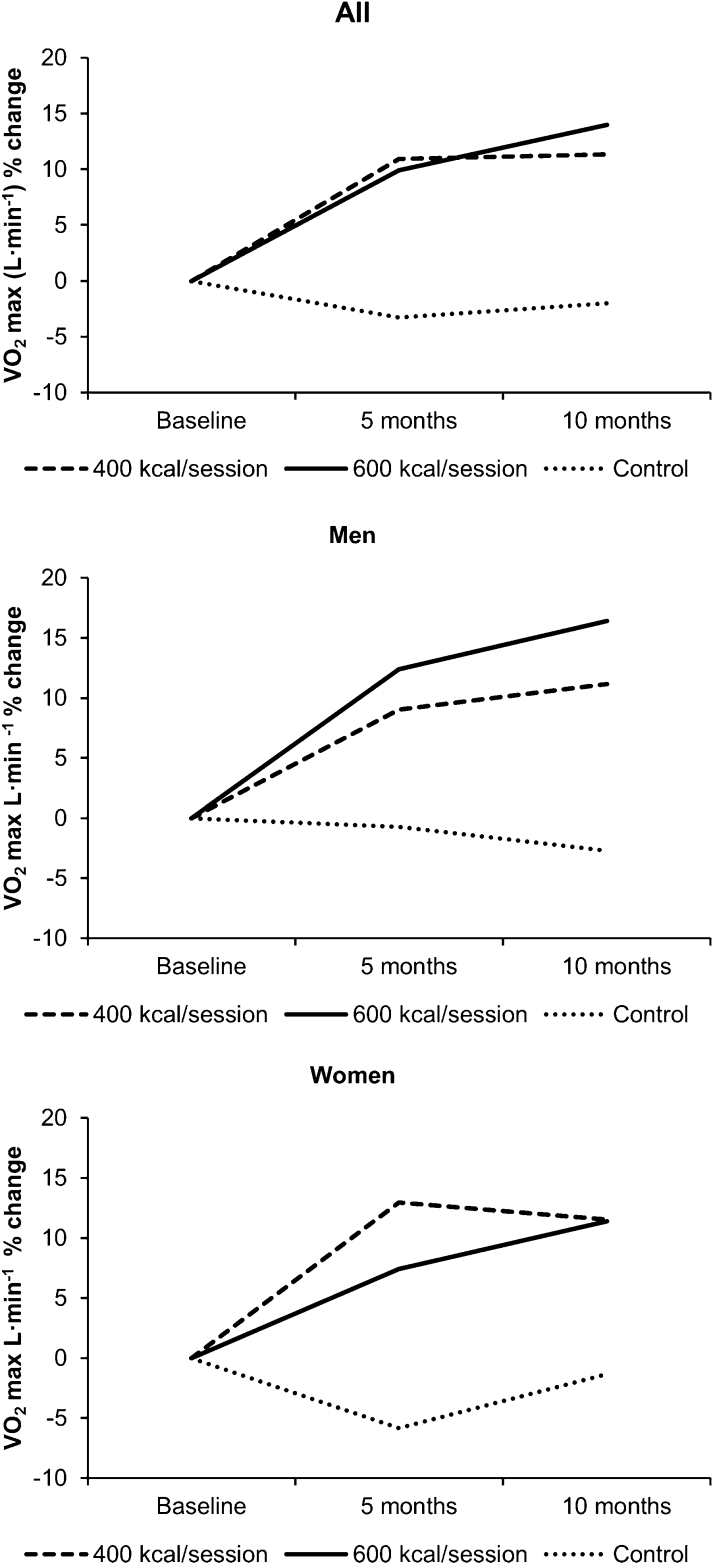
Percent change in VO_2_ max in the complete sample (*top*), men (*center*) and women (*bottom*)

VO_2_ max (L min^−1^) increased significantly in both the 400 (11.3 %) and 600 kcal session^−1^ groups (14 %) compared to control (−2.0 %; *p* < 0.001); however, the differences between exercise groups were not significant (Table 3; Fig. 1). Similar results were noted for change in relative VO_2_ max (mL kg^−1^ min^−1^); however, the magnitude of change was greater than for absolute VO_2_ max (L min^−1^) (400 group = 18.3 %; 600 group = 20.2 %) due to loss of body weight over the 10-mo intervention in both exercise groups.

**Table 3:** Change in cardiovascular fitness over the 10 mo intervention by group and gender

	Month	400 kcal session^−1^	600 kcal session^−1^	Control
*Total sample*				
VO_2_ max (L min^−1^)*	0	3.0 ± 0.7	3.1 ± 0.8	2.8 ± 0.6
	5	3.3 ± 0.8	3.4 ± 1.0	2.7 ± 0.6
	10	3.4 ± 0.8	3.5 ± 0.9	2.7 ± 0.5
VO_2_ max (ml kg^−1^ min^−1^)*	0	33.4 ± 6.6	34.2 ± 5.7	32.3 ± 5.0
	5	37.8 ± 7.1	38.0 ± 9.2	31.0 ± 5.8
	10	39.3 ± 7.9	40.9 ± 7.2	31.4 ± 5.3
*Men*				
VO_2_ max (L min^−1^)*	0	3.6 ± 0.5	3.7 ± 0.7	3.2 ± 0.4
	5	3.9 ± 0.5	4.1 ± 0.8	3.2 ± 0.4
	10	4.0 ± 0.5	4.2 ± 0.7	3.2 ± 0.4
VO_2_ max (ml kg^−1^ min^−1^)*	0	37.1 ± 6.5	36.4 ± 6.4	34.2 ± 5.8
	5	41.4 ± 7.6	41.6 ± 7.3	33.6 ± 6.7
	10	42.9 ± 8.0	44.2 ± 7.6	33.0 ± 6.4
*Women*				
VO_2_ max (L min^−1^)*	0	2.5 ± 0.4	2.5 ± 0.4	2.4 ± 0.2
	5	2.7 ± 0.4	2.7 ± 0.8	2.3 ± 0.3
	10	2.7 ± 0.5	2.8 ± 0.4	2.3 ± 0.3
VO_2_ max (ml kg^−1^ min^−1^)*	0	29.6 ± 4.2	31.8 ± 3.7	30.2 ± 3.3
	5	34.3 ± 4.6	34.3 ± 9.7	28.5 ± 3.7
	10	37.7 ± 6.2	37.3 ± 4.7	29.8 ± 3.7

Values are mean ± standard deviation

* Change in both the 400 and 600 kcal session^−1^ groups were significantly greater than control (p < 0.05). The differences for change between the 400 and 600 kcal session^−1^ groups were not statistically significant

The pattern of change in VO_2_ max (L min^−1^) shown in the total sample was also observed in both men and women (Table 3; Fig. 1). VO_2_ max increased significantly in both the 400 kcal session^−1^ (men = 11.1 %, *p* = 0.001; women = 11.4 %, *p* = 0.008) and 600 kcal session^−1^ groups (men = 16.4 %, *p* < 0.001; women = 11.4 %, *p* = 0.012) compared to control (men = −4.0 %, NS; women = −1.5 %, NS) with no significant differences for change in VO_2_ max between exercise groups (men *p* = 0.297; women p = 1.0). Similar results were noted for change in relative VO_2_ max (mL kg^−1^ min^−1^) by sex. However, the magnitude of change was greater for relative compared with absolute VO_2_ max (L min^−1^) due reductions in body weight in both men (400 = −3.8 kg, 600 = −5.9 kg) and women (400 = −4.1 kg, 600 = −4.4 kg). VO_2_ max (mL kg^−1^ min^−1^) increased 15.5 and 22.6 % in men in the 400 and 600 kcal session^−1^ groups, respectively and 21 and 17.7 % in women in the 400 and 600 kcal session^−1^ groups, respectively.

Results from secondary mixed modeling analysis indicated that when age, sex, baseline BMI, and baseline VO_2_ max are included in the model, neither exercise group or the mean number of exercise minutes per session were significant predictors of the change in aerobic fitness (Table 4). Mixed modeling also indicated that the change in VO_2_ max was greater in men than in women, and greater in those with lower VO_2_ max at baseline.

**Table 4:** Factors associated with change in VO_2_ max (L min^−1^) including either exercise group (A) or exercise min session^−1^ (B) as independent variables

	Estimate	*SE*	*p*
*A: Exercise Group* (400/600 kcal session^−1^)			
Intercept	47.7	9.2	<0.001
Age	0.2	0.3	0.47
Gender			
Men	14.7	3.8	<0.001
Women (reference)	–	–	–
Baseline BMI	−0.3	0.3	0.31
Baseline VO_2_ max (L min^−1^)	−10.4	2.7	<0.001
Exercise group			
400 kcal session^−1^ 600 kcal session^−1^ (reference)	−2.8	2.5	0.25
*B: Exercise* (min session^−1^)			
Intercept	71.1	17.6	<0.001
Age	0.2	0.3	0.55
Gender			
Men	−12.8	3.9	0.02
Women (reference)	–	–	–
Baseline BMI	0.0	0.0	0.58
Baseline VO_2_ max (L min^−1^)	−10.8	3.1	<0.001
Mean exercise (min session^−1^)	−0.1	0.1	0.30

### Other outcomes

Weight change over the 10 mo intervention in both the 400 (−4.3 %) and 600 kcal session^−1^ groups (−5.7 %) was significantly different than control; however, weight change between exercise groups did not differ significantly. There were no significant differences for weight change between men and women in either the 400 kcal session^−1^ (men = −3.7 %; women = −4.9 %) or 600 kcal session^−1^ groups (men = −5.9 %; women = −5.4 %) Energy intake (kcal d^−1^) was not different between exercise groups over the 10 mo intervention (see Washburn et al. 2015). Daily physical activity (including exercise and non-exercise activity) in both exercise groups was significantly higher than control, and did not change over the 10 mo intervention in either exercise group or in controls (for a detailed analysis, see Willis et al. (2014).

## Discussion

The association between aerobic fitness and health parameters is well established (Goodrich et al. 2012; Farrell et al. 2010; Fogelhom 2010; Lee et al. 2012; DeFina et al. 2015). However, there are limited data regarding the association between exercise dose (volume and intensity) and changes in aerobic fitness in overweight and obese young adults, a group for which exercise may be an attractive option to reduce chronic disease risk factors including body weight. The results of this 10-mo randomized trial suggest that increased volume of vigorous intensity exercise was not associated with increased aerobic fitness.

Our results are in agreement with previous reports which have shown no effect of exercise volume at fixed intensities, ranging from 45 to 80 % VO_2_ max, on aerobic fitness (Duncan et al. 2005; Asikainen et al. 2002; Hautala et al. 2003; Rosenkilde et al. 2012). Similar to the present study, Rosenkilde and colleagues utilized aerobic exercise in a study of overweight young men (mean age and BMI = 29 y and 28 kg m^−2^) comparing 300 and 600 kcal d^−1^ (Rosenkilde et al. 2012). However, their study only lasted 13 wk in duration with 10 wk of intervention. Although their exercise prescription, study design, and rates of adherence (99 and 96 %) were similar to the present study, supervision of exercise during their intervention was unclear. Participants were asked to exercise at ≥70 % VO_2_ max 3 d wk^−1^ and were permitted to self-select their intensity the other days, leading to average estimated intensities of ~66–67 % VO_2_ max derived from heart rate data. Participants exercised an average of 6 d wk^−1^ for 29.9 and 55.2 min, with EEEx averaging 335 and 653 kcal d^−1^, respectively. Therefore, estimated weekly EEEx was equal to 2010 and 3918 kcal wk^−1^ and weekly exercise time was 179.4 and 331 min wk^−1^. Reductions in body weight were modest (−4 and −3 % in the 300/600 kcal groups, respectively) and not significantly different between groups. Changes in relative VO_2_max were also not different between groups (18 and 17 % increases, respectively), thus agreeing with our results that increased exercise volume, when intensity is matched, does not result in greater improvements in VO_2_ max.

In contrast to the results of the current study, some investigators have concluded that the amount of exercise may be more important than intensity for eliciting increases in aerobic fitness (Church et al. 2007; Duscha et al. 2005; Dalleck et al. 2009). For example, Church et al. (2007) conducted a 6 mo supervised exercise intervention in overweight and obese older women (~57 years) randomly assigned to exercise at 50 % VO_2_ peak, 3–4 d wk^−1^ at one of 3 amounts (4, 8, or 12 kcal kg^−1^ wk^−1^.) or a non-exercise control. Results showed the exercise energy expenditure and minutes of exercise actually completed were: 4 kcal kg^−1^ group = 335 kcal wk^−1^, 72 min wk^−1^; 8 kcal kg^−1^ group = 681 kcal wk^−1^, 136 min wk^−1^; 12 kcal kg^−1^ group = 1006 kcal wk^−1^, 192 min wk^−1^. A graded dose–response change in aerobic fitness across exercise groups was observed. Compared with the non-exercise control group, VO_2_ max increased 4.2, 6.0 and 8.2 % in the 4, 8 and 12 kcal kg^−1^ groups, respectively. Duscha et al. (2005) analyzed data on the change in aerobic fitness from the Studies of Targeted Risk Reduction Intervention Through Defined Exercise (STRRIDE) trial and their results indicated that the increase in VO_2_ max (L min^−1^) in the high amount group (15.4 %) was significantly greater than in the low amount group (11.5 %).

Differences in baseline aerobic fitness and exercise volume assigned to the lowest volume group may at least partially explain the discrepant results of studies evaluating the effect of exercise volume at fixed intensities on changes in aerobic fitness. Both the level of baseline aerobic fitness and the volume of prescribed exercise in the lowest volume group are lower in studies which conclude that exercise amount has a significant impact on changes in aerobic fitness. For example, the average baseline VO_2_ max in studies supporting the importance of exercise volume (Church et al. 2007; Duscha et al. 2005; Dalleck et al. 2009) was 22.2 mL kg^−1^ min^−1^ (range 15.3–29.2) compared with 33.2 mL kg^−1^ min^−1^ (range 23–43) in studies (including the present study) which found no effect of exercise volume on aerobic fitness (Duncan et al. 2005; Asikainen et al. 2002; Hautala et al. 2003; Rosenkilde et al. 2012). Therefore, increased exercise volume may induce increased aerobic fitness only among individuals with initially low level of aerobic fitness at baseline. Our mixed modeling results, which indicated greater increases in fitness among participants with lower baseline VO_2_ max, offer partial support for this explanation. In addition, in studies which support the importance of exercise volume in increasing aerobic fitness, the average volume in the lowest volume group was 115 min wk^−1^ (range 72–149 min wk^−1^) compared with 198 min wk^−1^ (range 180–331 min wk^−1^) in studies which found no effect of exercise volume on aerobic fitness. For example, Church et al. (2007) compared exercise at 50 % VO_2_ peak across 3 volumes: 72, 136 and 192 min wk^−1^. The lowest exercise volume (72 min wk^−1^) is less than the minimal recommendations for increasing aerobic fitness (Garber et al. 2011). Thus, the finding of a dose–response for exercise volume in these studies may be an artifact of comparing a low exercise volume, which would be expected to induce minimal increases in aerobic fitness, with a higher volume of exercise where substantial increases in aerobic fitness are expected. Finally, it is also worth noting that the participants of the studies showing a dose response were considerably older than the studies that have failed to show a response (average = 55.6 vs. 32.3 years). Therefore it is possible that older individuals with lower levels of aerobic fitness compared to their younger peers more favorably responded to distinct changes in volume.

MET-2 was an efficacy study in which exercise dose was tightly controlled and all exercise sessions were completed under supervision. Exercise dose was prescribed by energy expenditure rather than time and the dose was delivered with a high degree of precision (±1 % of target). Previous studies have dosed exercise by energy expenditure (Church et al. 2007; Asikainen et al. 2002; Rosenkilde et al. 2012); however, EEEx was not assessed in these studies, therefore the precise exercise dose was not known. Our results suggest that increased exercise volume, measured as both energy expenditure and time, was not associated with increased aerobic fitness. Only two previous studies on exercise volume and aerobic fitness have included both men and women (Duscha et al. 2005; Duncan et al. 2005); however, data were not presented by sex. The results of MET-2 suggest that increased exercise volume does not increase aerobic fitness in either men or women. The sample for MET-2 was limited to otherwise healthy, sedentary, overweight or obese young adults. Thus, the generalizability of our results, to samples with other demographic characteristics, is unknown. However, individuals with characteristics similar to our study sample represent a sizeable segment of the US population who would likely benefit from exercise as evidenced by current estimates for overweight and obesity among men (~67 %) and women (~56 %) age 20–39 years (Flegal et al. 2012; Ogden et al. 2014).

In conclusion, our results indicate no difference for change in aerobic fitness between exercise at ~80 % HR max over 10 mo at either 2000 or 3000 kcal wk^−1^. Participants completing the 2000 kcal wk^−1^ protocol increased VO_2_ max by 11.3 and ~46 % of the sample lost ≥5 % of baseline body weight (Donnelly et al. 2013). Epidemiologic studies have suggested that each 1-MET increase in aerobic fitness confers an 8 to 19 % reduction in cardiovascular disease and all-cause mortality (Myers et al. 2002; Gulati et al. 2003; Lee et al. 2011). Based on the observed increase in aerobic fitness alone (1.7 METs), participants in the current study may expect a 14–32 % decrease in mortality risk. An exercise prescription of 2000 kcal wk^−1^ which required exercise (5 d wk^−1^) of approximately 31 min d^−1^ for men and 48 min d^−1^ for women may represent a reasonable recommendation for improving health and reducing mortality risk in overweight and obese young adults.

## Abbreviations

BMI: body mass index
HR: heart rate
VO_2_: oxygen consumption
EEEx: exercise energy expenditure
MET-2: Midwest Exercise Trial 2
MET: metabolic equivalent
RPE: rating of perceived exertion
RER: respiratory exchange ratio
NS: non-significant
kcal: kilocalorie
d: day
wk: week
mo: month
